# Immunomodulatory Effects of Calcium and Strontium Co-Doped Titanium Oxides on Osteogenesis

**DOI:** 10.3389/fimmu.2017.01196

**Published:** 2017-09-29

**Authors:** Xiangwei Yuan, Huiliang Cao, Jiaxing Wang, Kaiwei Tang, Bin Li, Yaochao Zhao, Mengqi Cheng, Hui Qin, Xuanyong Liu, Xianlong Zhang

**Affiliations:** ^1^Department of Orthopedics, Shanghai Sixth People’s Hospital, Shanghai Jiao Tong University, Shanghai, China; ^2^State Key Laboratory of High Performance Ceramics and Superfine Microstructure, Shanghai Institute of Ceramics, Chinese Academy of Sciences, Shanghai, China

**Keywords:** immunity, osteogenesis, calcium, strontium, macrophage polarization

## Abstract

The effects of calcium (Ca) or strontium (Sr) on host osteogenesis and immune responses have been investigated separately. In clinical practice, these two elements may both be present around an orthopedic device, but their potential synergistic effects on osteogenesis and the immune response have not been explored to date. In this work, we investigated the immunomodulatory effects of Ca and Sr co-doped titanium oxides on osteogenesis *in vitro* using the mouse macrophage cell line RAW 264.7 alone and in co-culture with mouse bone mesenchymal stem cells (BMSCs), and *in vivo* using a mouse air-pouch model. Coatings containing Ca and Sr at different concentration ratios were fabricated on titanium substrates using micro-arc oxidation and electrochemical treatment. The *in vitro* and *in vivo* results demonstrated that the Ca and Sr concentration ratio has a marked influence on macrophage polarization. The coating with a Ca/Sr ratio of 2:1 was superior to those with other Ca and/or Sr concentrations in terms of modulating M2 polarization, which enhanced osteogenic differentiation of mouse BMSCs in co-culture. These findings suggest that the osteoimmunomodulatory effect of a titanium-oxide coating can be enhanced by modulating the concentration ratio of its components.

## Introduction

The immune system plays an important role in tissue repair and reconstruction, and is closely linked with the skeletal system. Moreover, bone formation is regulated to an extent by the immune system; this is known as osteoimmunomodulation (1, 2). This effect may account for the inconsistent osteogenic capacity of biomaterials *in vitro* and *in vivo*. Accordingly, modulation of the nature, duration, and magnitude of the host immune response could enhance osseointegration of titanium (Ti) implants.

Macrophages play a regulatory role in the host–implant interaction. Following implant placement, monocytes in bone marrow are chemoattracted to the biomaterial site and gradually differentiate into macrophages, which are polarized into one of two phenotypes due to the local micro-environmental conditions (3, 4). These are the M1 (classically activated/inflammatory) and M2 (alternatively activated/regenerative) macrophage phenotypes, similar to Th1 and Th2 T-helper cells (4, 5). M1 macrophages are pro-inflammatory and exert an immunostimulatory effect, while M2 macrophages are anti-inflammatory and promote tissue repair (4, 6–9). M1 macrophages produce an array of inflammatory mediators, such as tumor necrosis factor-α (TNF-α), and these mediators induce osteoclasts to resorb bone, possibly leading to aseptic loosening of implants (10). M2 macrophages enhance osteogenesis by expressing and secreting pro-osteogenic factors, such as bone morphogenetic protein 2 (BMP2), transforming growth factor-β (TGF-β), and vascular endothelial growth factor (VEGF), which contribute to the osteogenic differentiation of bone mesenchymal stem cells (BMSCs) (11–14). Thus, induction of an appropriate macrophage phenotype is important in patients with biomaterial implants. Furthermore, focusing on osteoblastic lineage cells while ignoring the role of macrophages would hamper evaluation of the host–implant interaction.

Biomaterial implants can induce macrophage polarization by modifying their porosity, pore size, surface topography and chemistry, and active components (15–17). Because active components—such as Ca^2+^, Sr^2+^, and Mg^2+^—mediate human chemobiological homeostasis, they may be capable of modulating macrophage polarization. These active elements can induce a switch from M1 to M2, and downregulate the production of pro-inflammatory cytokines (TNF-α and IL-6) and upregulate the production of growth factors (BMP2, VEGF, and TGF-β) by M2 macrophages to enhance osteogenic differentiation of BMSCs (16, 18–20). However, most studies have focused on the effects of a single element, and synergistic or competitive effects among the cations were neglected even though they were simultaneously presented, which is unfavorable for optimization of their osteoimmunomodulatory function. Ca and Sr are indispensable for health. Indeed, a Ca–Sr imbalance is implicated in several diseases (21, 22). For example, experimental animals fed large amounts of Sr developed rickets due to disruption of intestinal Ca absorption and synthesis of vitamin D (23). Moreover, a high dose of Sr reportedly reduces bone mineralization (24).

Titanium is used in orthopedics and dentistry due to its outstanding mechanical strength, biocompatibility, and resistance to corrosion (17, 25, 26). However, undesirable immune responses to Ti and its alloys may result in poor osseointegration, implant loosening, or premature failure (27, 28). In this study, the effects on macrophage polarization of coatings containing Ca and Sr at various concentration ratios on Ti substrates were investigated. A coating containing Ca and Sr at a 2:1 ratio increased M2 macrophage polarization, which enhanced osteogenic differentiation of mouse BMSCs.

## Materials and Methods

### Material Fabrication and Characterization

Commercial pure Ti was cut into square plates (10 mm × 10 mm × 1 mm or 20 mm × 20 mm × 1 mm), which were polished with 1000# abrasive paper, ultrasonically cleaned in ethanol and micro-arc oxidized (MAO) in an electrolyte solution containing 5.5 g/L glycerophosphate disodium salt pentahydrate (C_3_H_7_Na_2_O_6_P⋅5H_2_O; Kelong, China) and 5.0 g/L sodium metasilicate non-ahydrate (Na_2_SiO_3_⋅9H_2_O; Sinopharm, China) to fabricate a porous titanium-oxide layer. To load Sr and/or Ca into the surface layer, the MAO-treated plates were electrochemically treated (ECT) in solutions of calcium chloride (CaCl_2_; Sinopharm) and strontium dichloride (SrCl_2_⋅6H_2_O; Sinopharm) at various concentration ratios by applying a negative potential (0.8 A/cm^2^) (a graphite plate was used as the counter-electrode) for 15 min (Table 1). Endotoxin contamination was detected by Tachypleus Amebocyte Lysate assay (TAL; Zhanjiang A & C Biological Ltd., China), which has a sensitivity of 10–0.01 endotoxin units (EU)/mL.

**Table 1:** The sample groups concerned in this study.

Group name	Treatment history
Titanium (Ti)	Commercial pure Ti was polished with 1000# abrasive paper
Ti-Ca10	The Ti group was further MAO treated, then electrochemically treated (ECT) in a solution with 10 g/L CaCl_2_
Ti-Sr10	The Ti group was further MAO treated, then ECT in a solution with 10 g/L SrCl_2_⋅6H_2_O
Ti-Ca10Sr10	The Ti group was further MAO treated, then ECT in a solution with 10 g/L CaCl_2_ and 10 g/L SrCl_2_⋅6H_2_O
Ti-Ca10Sr5	The Ti group was further MAO treated, then ECT in a solution with 10 g/L CaCl_2_ and 5 g/L SrCl_2_⋅6H_2_O
Ti-Ca10Sr2.5	The Ti group was further MAO treated, then ECT in a solution with 10 g/L CaCl_2_ and 2.5 g/L SrCl_2_⋅6H_2_O

Sample surface morphology was visualized by scanning electron microscopy (SEM; JEOL JSM-6700F, Japan), and the chemical states of Ca and Sr were determined by X-ray photoelectron spectroscopy (XPS; Axis UltraDLD, Japan). As described previously (29), to assess Ca and Sr release kinetics, samples were immersed in 10 mL sterile 0.9% saline at 37°C without stirring for 7, 14, 21, and 28 days, and Ca and Sr in solution were quantified by inductively coupled plasma atomic emission spectrometry (ICP-AES; Varian, Inc., Palo Alto, CA, USA). Sample wettability was determined by measuring the contact angle of deionized water (2 µL).

### *In Vitro* Experiments

#### RAW264.7 Cell Culture

Mouse RAW264.7 macrophages were purchased from the Type Culture Collection of the Chinese Academy of Sciences (Shanghai, China). RAW264.7 cells were cultured in Dulbecco’s Modified Eagle’s Medium (DMEM; HyClone, USA) with 10% heat-inactivated fetal bovine serum (FBS; Gibco, USA) and 1% penicillin/streptomycin (HyClone). RAW264.7 cells (1 × 10^6^) were seeded onto the surfaces of samples in six-well culture plates containing DMEM and incubated at 37°C in 5% CO_2_ unless indicated otherwise. Medium was refreshed every 2 days.

#### Cell Viability Assay

Live/dead staining was performed to assess the viability of RAW264.7 cells. After 4 days of culture on samples, RAW264.7 cells were twice rinsed gently with phosphate-buffered saline (PBS; pH 7.4), stained using a live/dead kit (Invitrogen, Carlsbad, CA, USA) for 15 min, and visualized by fluorescence microscopy (Olympus, Japan).

#### Cell Proliferation Assay

A cell counting kit-8 (CCK-8) (Dojindo, Japan) was used to assess RAW264.7 cell proliferation. After 2 and 6 days of culture on samples, RAW264.7 cells were twice rinsed gently with PBS and incubated for 4 h with fresh complete medium and CCK-8 at a 10:1 (v/v) ratio at 37°C. The supernatants were transferred to a new 96-well plate (200 µL per well), and the absorbance at a wavelength of 450 nm was determined.

#### Flow Cytometry

Expression of the RAW264.7 cell-surface markers cluster of differentiation 206 (CD206; M2 marker) and C-C chemokine receptor type 7 (CCR7; M1 marker) was determined by flow cytometry. After 4 days of culture on samples, RAW264.7 cells were collected, centrifuged at 1,200 rpm for 5 min at 4°C, resuspended in PBS containing 1% bovine serum albumin (BSA) to block Fc-receptors for 30 min at room temperature, and incubated with phycoerythrin (PE)-conjugated anti-mouse CD206 and allophycocyanin (APC)-labeled anti-mouse CCR7 (eBioscience) antibodies for 1 h at room temperature in the dark. PE-labeled IgG2a and APC-labeled IgG2a (eBioscience) were used as negative controls. The cells were washed three times in PBS containing 1% BSA and transferred to FACS tubes (200 µL per tube) for determination using a Guava easyCyte™ HT flow cytometer (Millipore, Billerica, MA, USA); 5,000 events per tube were analyzed. Results were processed using guavaSoft 3.1.1 software.

#### Immunofluorescence Staining

Expression of the M2 marker CD206 and the M1 marker CCR7 in RAW264.7 cells was assayed by immunofluorescence. After 4 days of culture on samples, RAW264.7 cells were fixed in 4% paraformaldehyde for 30 min at room temperature, rinsed three times with PBS, and resuspended in PBS containing 1% BSA to block Fc-receptors for 30 min at room temperature. Next, the cells were incubated with primary antibodies against CD206 and CCR7 (Abcam, Cambridge, UK) overnight at 4°C. Cells were incubated with goat anti-rat Alexa Fluor 488 (1:200) and goat anti-rabbit Alexa Fluor 594 (1:200; Abcam) secondary antibodies for 1 h and nuclei were stained with 46-diamidino-2-phenylindole (DAPI) for 5 min at room temperature in the dark. Finally, cells were visualized and enumerated by fluorescence microscopy (Olympus, Japan).

#### Enzyme-Linked Immunosorbent Assay (ELISA)

The concentrations of BMP2 (pro-osteogenic), VEGF (pro-angiogenic), TNF-α (pro-inflammatory), and interleukin-10 (IL-10; anti-inflammatory) in RAW264.7 cell culture medium were determined by ELISA (eBioscience). After 4 days of culture on samples, RAW264.7 cell supernatants were collected and the absorbance at 450 nm was determined using a microplate reader. The concentrations of the abovementioned factors were calculated using standard curves.

#### Real-time Polymerase Chain Reaction (RT-PCR)

RT-PCR was used to quantify the expression of CD206, CCR7, BMP2, and VEGF in RAW264.7 cells using glyceraldehyde 3-phosphate dehydrogenase (GAPDH) as a control. The forward and reverse primers are listed in Table 2. After 4 days of culture of RAW264.7 cells on samples, total RNA was extracted using TRIzol reagent (Invitrogen). Complementary DNA (cDNA) was synthesized from 1 µg of total RNA using a RevertAid First Strand cDNA Synthesis kit (Thermo). Gene expression was quantified using FastStart Universal SYBR Green Master Mix (Rox, Roche) and a PCR instrument (ABI). Expression levels of target genes were evaluated by the 2^−ΔΔCt^ method and were normalized to the mean threshold cycle (Ct) value of GAPDH.

**Table 2:** Primers for real-time polymerase chain reaction (RT-PCR) used in this study.

Gene	Primer sequences (F, forward; R, reverse; 5′−3′)	Length(bp)
GAPDH	F:AGGAGCGAGACCCCACTAACA	247
	R:AGGGGGGCTAAGCAGTTGGT	
β-actin	F:GTGACGTTGACATCCGTAAAGA	287
	R:GTAACAGTCCGCCTAGAAGCAC	
CD206	F:TACTTGGACGGATAGATGGAGG	230
	R:CATAGAAAGGAATCCACGCAGT	
CCR7	F:GGTGGCTCTCCTTGTCATTTTC	264
	R:AGGTTGAGCAGGTAGGTATCCG	
VEGF	F:AGGAGTACCCCGACGAGATAGA	198
	R:CACATCTGCTGTGCTGTAGGAA	
BMP2	F:AACGAGAAAAGCGTCAAGCC	119
	R:AGGTGCCACGATCCAGTCAT	
RUNX2	R:AGCGGACGAGGCAAGAGTTT	219
	F:AGGCGGGACACCTACTCTCATA	
PPARγ	R:GAGGCAGATGACCTGGAAAGT	312
	F:TGCGTGAACTCCGTAGTGGTA	

#### Western Blotting

Western blotting was performed to quantify CD206, CCR7, VEGF, and BMP2 protein levels. After 4 days of culture on samples, RAW264.7 cells were lysed with lysis buffer, and protein levels were quantified using a bicinchoninic acid (BCA) kit (Servicebio Technology Co., Ltd.). Proteins were resolved by sodium dodecyl sulfate-polyacrylamide gel electrophoresis, and transferred to nitrocellulose membranes. Membranes were blocked for 1 h in Tris-buffered saline (TBS)-Tween 20 buffer containing 5% (w/v) non-fat milk, incubated with primary antibodies against CD206, CCR7, VEGF, BMP2, and β-actin (1:1,000; Servicebio Technology Co., Ltd.) overnight at 4°C, rinsed three times with TBS-Tween 20, and incubated with horseradish peroxidase-conjugated secondary antibodies for 1 h at room temperature. After rinsing three times in TBS-Tween 20, protein bands were visualized using alpha EaseFC (Alpha Innotech, San Leandro, CA, USA) in a dark room. The intensity of the protein bands was quantified using Adobe Photoshop software.

#### BMSC Isolation and Culture

BMSCs were isolated and cultured as described previously (30). Briefly, primary cells were isolated from the femurs and tibiae of 6-week-old male C57BL/6 mice under sterile conditions and cultured for 4 days in DMEM containing 10% FBS and 1% penicillin/streptomycin at 37°C and 5% CO_2_. Non-adherent cells were rinsed off and the medium was refreshed. The remaining adherent primary BMSCs were named P_0_. BMSCs were expanded after reaching 80–90% confluence. P_3_ BMSCs were used in subsequent experiments.

#### Co-Culture of BMSCs and RAW264.7 Cells

Transwell^®^ culture plates were used for co-culture of BMSCs and RAW264.7 cells (31). Briefly, RAW264.7 cells were cultured on samples in complete medium for 4 days, then transferred to a 24-well culture plate with an 8-µm-pore-size filter containing complete medium (0.5 × 10^4^ per well) and incubated for 2 h. Next, BMSCs (1 × 10^4^ per well) were added to the Transwell^®^ plate; this enabled culture of BMSCs and RAW264.7 cells in the same medium without direct contact. BMSCs were also exposed to the conditioned medium of RAW264.7 cells. All incubations were performed at 37°C in 5% CO_2_.

#### Extracellular Matrix (ECM) Mineralization Assay

Extracellular matrix mineralization was evaluated by Alizarin red staining. After co-culture for 21 days, BMSCs were fixed in 4% formaldehyde for 30 min and stained with Alizarin red for 5 min. Cells were rinsed gently three times in PBS and visualized by optical microscopy. Cetylpyridinium chloride (10%) in 10 mM sodium phosphate was applied to elute the bound stain, and the optical densities (ODs) at 600 nm of the eluents were determined.

#### Alkaline Phosphatase Activity (ALP) Assay

The effect of co-culture with RAW264.7 cells on BMSC differentiation was assessed by assaying ALP. After co-culture for 14 days, BMSCs were lysed with 0.1% Triton X-100 for 30 min at room temperature, and the supernatants were incubated with p-nitrophenyl phosphate (Sigma-Aldrich, St. Louis, MO, USA) for 30 min at 37°C. The ODs at 405 nm of the supernatants were determined, and total protein contents were determined by BCA protein assay (Servicebio Technology Co., Ltd.). ALP activity is expressed as optical density (OD, 405 nm) per milligram total protein.

#### Expression of Osteogenic and Adipogenic Genes

Real-time polymerase chain reaction was used to quantify the expression of osteogenic [BMP2 and runt-related transcription factor 2 (RUNX2)] and adipogenic [peroxisome proliferator-activated receptor γ (PPARγ)] genes (Table 2). Expression levels were normalized to that of β-actin. After co-culture for 14 days, gene expression was assayed using the methods described above.

### *In Vivo* Experiments

#### Mouse Air-Pouch Model

Six-week-old male pathogen-free C57BL/6 mice were maintained under specific pathogen-free conditions at our animal care facility. Animal maintenance and procedures were conducted according to the policy of the Institutional Animal Care and Use Committee of Shanghai Jiao Tong University, the regulations for the Administration of Affairs Concerning Experimental Animals (China, 2014), and the National Institutes of Health Guide for the Care and Use of Laboratory Animals (GB14925-2010). Animal experiments were approved by the Animal Care and Experiment Committee of Shanghai Sixth People’s Hospital, which is affiliated with Shanghai Jiao Tong University. The mouse air-pouch model was described previously (32, 33). Briefly, 4 mL of sterile air was injected subcutaneously into the lower dorsal area of mice, resulting in formation of a dorsal air-pouch. Four days later, a second injection of 4 mL sterile air was performed to reinforce the air-pouches. Mice were anesthetized 24 h later by intraperitoneal injection of 4% chloral hydrate (0.4 mL per 100 g body weight), and the skin over the air-pouch was shaved thoroughly. Under sterile conditions, a surgical incision was made from the upper margin of the air-pouch, a material sample was implanted into the air-pouch, the skin was disinfected, and the surgical incision was sutured.

#### Air-Pouch Exudates and Tissues

Seven days after sample implantation, mouse air-pouch exudates and tissues were collected. Briefly, mice were anesthetized and air-pouches were washed by repeated injections of 3 mL PBS using a sterile syringe. Exudates (1.5 mL) were centrifuged at 1,200 rpm for 5 min at 4°C. The supernatants were stored at −80°C for ELISA, and the pellets were used for flow cytometry. The air-pouch tissue (including the sample) was fixed in 4% formaldehyde for histological analysis. Finally, mice were euthanized by cervical dislocation under general anesthesia.

#### Flow Cytometric Analysis of Air-Pouch Exudates

Cell pellets from the air-pouch exudates were resuspended in PBS containing 1% BSA to block Fc-receptors for 30 min at 37°C and incubated with fluorescein isothiocyanate (FITC)-labeled anti-mouse F4/80, APC-labeled anti-mouse CCR7, and PE-labeled anti-mouse CD206 antibodies (eBioscience) for 1 h at room temperature in the dark. Corresponding isotype controls were also established. Finally, cells were analyzed using the methods described above.

#### Determination of TNF-α and IL-10 Levels in Air-Pouch Exudates

Tumor necrosis factor-α and IL-10 levels in air-pouch exudate supernatants were quantified using ELISA kits (eBioscience) according to the manufacturer’s instructions.

#### Histological Analysis of Air-Pouch Tissues

Air-pouch tissues were subjected to hematoxylin and eosin (HE) and Masson’s trichrome staining. Air-pouch tissues were fixed in 4% formaldehyde for 24 h, embedded in paraffin wax, and sectioned at 4 µm. After dewaxing and hydration, sections were stained with HE and Masson’s trichrome. Stained sections were visualized by optical microscopy. Image Pro Plus software was used to evaluate fibrous capsule thickness and the number of infiltrating cells in five random locations. Other air-pouch sections were incubated in 3% H_2_O_2_ for 10 min after dewaxing and hydration, and subjected to immunofluorescence staining as above.

### Statistical Analysis

SPSS 17.0 software was used for statistical analyses. Quantitative data are expressed as mean ± SD. One-way analysis of variance and the Student–Newman–Keul’s *post hoc* test were used to determine the significance of differences. A value of *p* < 0.05 was considered to indicate a significant difference.

## Results

### Material Characterization

**Figure 1:**
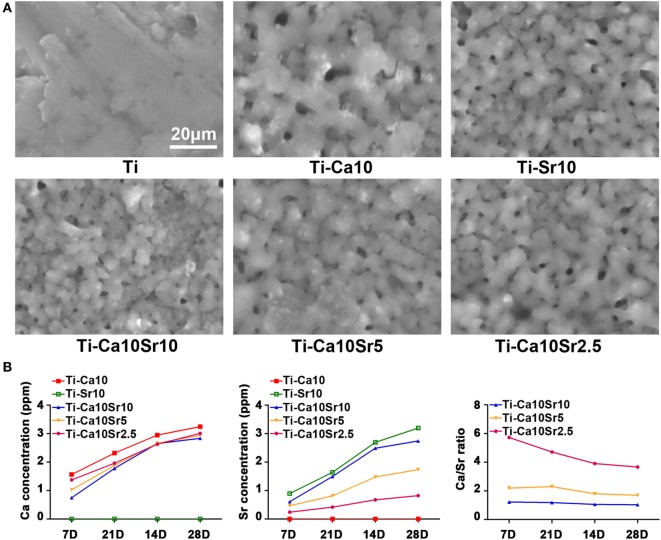
**(A)** The surface morphology of different samples displayed by scanning electron microscopy. Scale bar, 20 µm. **(B)** Calcium (Ca) and strontium (Sr) releasing concentrations and ratios released from different samples at day 7, 14, 21, and 28.

Sample surface morphology was visualized by SEM (Figure 1A). The surface of pure Ti was flat, while the treated materials (designated Ti-Ca10, Ti-Sr10, Ti-Ca10Sr10, Ti-Ca10Sr5, and Ti-Ca10Sr2.5 according to ECT conditions; Table 1) exhibited porous surfaces. However, the surface chemistry of the treated materials differed, as revealed by the Ca and Sr release kinetics (Figure 1B). The Ca and Sr concentrations in solution increased gradually with increasing duration of immersion. During immersion, the Ca:Sr ratios of the Ti-Ca10Sr10 and Ti-Ca10Sr5 groups were maintained at 1:1 and 2:1, respectively, whereas the Ca:Sr ratio of the Ti-Ca10Sr2.5 group was 6:1, 5:1, and 4:1 on days 7, 14, and 21/28, respectively. The XPS spectra and water contact angles (Figure S1 in Supplementary Material) confirmed that sample surface properties differed according to Ca:Sr ratio.

### *In Vitro* Responses of RAW264.7 Cells

**Figure 2:**
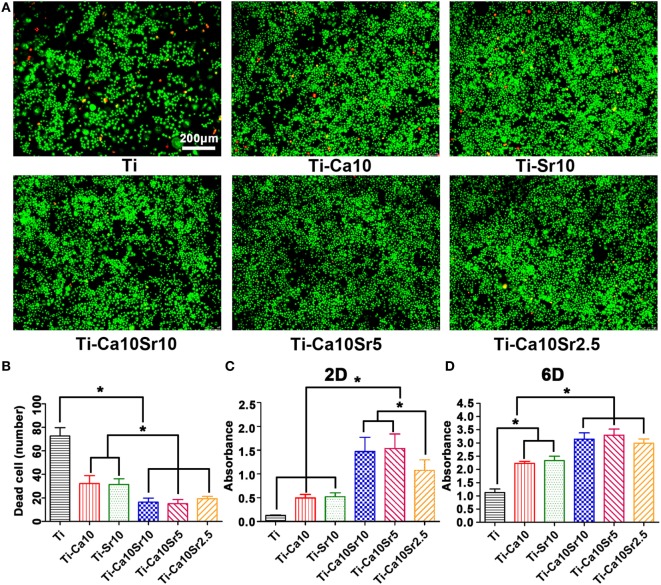
The viability and the proliferation of RAW264.7 cells on different samples were evaluated by live/dead staining and cell counting kit-8 (CCK-8) assay, respectively. **(A)** Fluorescent images of RAW264.7 cells cultured for 4 days, dead cells were stained in red and live cells in green. Scale bar, 100 µm. **(B)** Numbers of dead cells in the images of live/dead staining. **(C,D)** CCK-8 assay of RAW264.7 cells cultured for 2 and 6 days, respectively. *p < 0.05.

RAW264.7 cell viability was evaluated by live/dead staining, in which live and dead cells are stained green and red (Figure 2A). RAW264.7 cell viability exhibited the following trend: Ti-Ca10Sr10, Ti-Ca10Sr5, and Ti-Ca10Sr2.5 > Ti-Ca10; and Ti-Sr10 > Ti (Figure 2B). This was confirmed by determining the cell proliferation rates. At day 2, the Ti-Ca10Sr10 and Ti-Ca10Sr5 groups exhibited the highest cell proliferation rate (Figure 2C). The cell proliferation rates in the Ti-Ca10Sr10, Ti-Ca10Sr5, and Ti-Ca10Sr2.5 groups were significantly higher than those in the Ti-Sr10, Ti-Ca10, and Ti groups at day 6 (Ti-Ca10Sr10, Ti-Ca10Sr5, and Ti-Ca10Sr2.5 > Ti-Ca10; and Ti-Sr10 > Ti) (Figure 2D).

**Figure 3:**
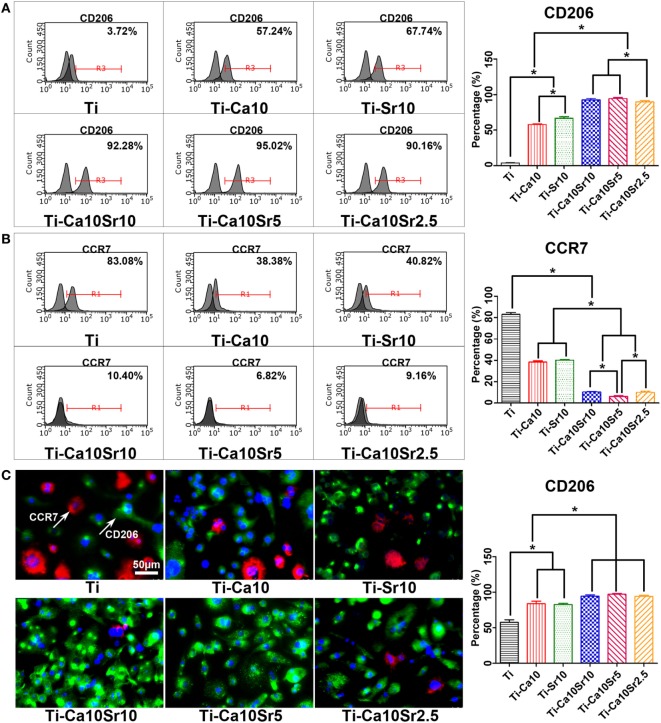
The polarization of RAW264.7 cells on different samples was evaluated by flow cytometry and immunofluorescence staining at day 4 of culture. **(A,B)** Representative histograms and quantitative analysis for RAW264.7 cells expressing cluster of differentiation 206 (CD206) (M2 maker) and C-C chemokine receptor type 7 (CCR7) (M1 maker), respectively, through flow cytometry. **(C)** Immunofluorescence images for CCR7 (M1 marker, stained in red) and CD206 (M2 marker, stained in green) of RAW264.7 cells; nuclei were stained in blue with 46-diamidino-2-phenylindole. Scale bar, 50 µm. And percentage of immunofluorescence-positive RAW264.7 cells expressing M2 maker CD206. *p < 0.05.

Polarization of RAW264.7 cells toward the M2 and M1 phenotypes was analyzed by flow cytometry for CD206 and CCR7, respectively. The proportion of M2 macrophages exhibited the following trend: Ti-Ca10Sr10 and Ti-Ca10Sr5 > Ti-Ca10Sr2.5 > Ti-Sr10 > Ti-Ca10 > Ti (Figure 3A). In contrast, the proportion of M1 macrophages showed the following trend: Ti > Ti-Ca10, and Ti- Sr10 > Ti-Ca10Sr10 and Ti-Ca10Sr2.5 > Ti-Ca10Sr5 (Figure 3B). Immunofluorescence staining for CD206 (green; M2 macrophages) and CCR7 (red; M1 macrophages) showed the following trend in the proportion of M2 macrophages: Ti-Ca10Sr10, Ti-Ca10Sr5, and Ti-Ca10Sr2.5 > Ti-Sr10; and Ti-Ca10 > Ti (Figure 3C).

**Figure 4:**
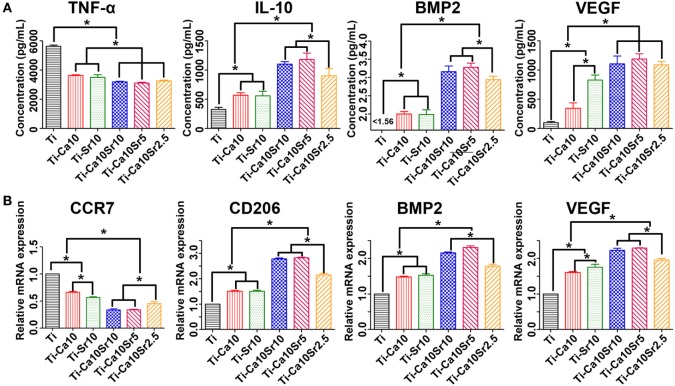
Enzyme-linked immunosorbent assay determination of cytokines, and real-time polymerase chain reaction analysis of genes from RAW264.7 cells at day 4 of culture on different samples. **(A)** The production of cytokines tumor necrosis factor-α (TNF-α), interleukin-10 (IL-10), bone morphogenetic protein 2 (BMP2), and vascular endothelial growth factor (VEGF), respectively. **(B)** The expression of genes C-C chemokine receptor type 7 (CCR7), cluster of differentiation 206 (CD206), BMP2, and VEGF, respectively. *p < 0.05.

Tumor necrosis factor-α, IL-10, BMP2, and VEGF production by RAW264.7 cells was determined by ELISA (Figure 4A). The TNF-α concentration in culture supernatant showed the following trend: Ti > Ti-Sr10 and Ti-Ca10 > Ti-Ca10Sr10, Ti-Ca10Sr5, and Ti-Ca10Sr2.5. By contrast, the IL-10 concentration in the Ti-Ca10Sr5 group was ~3.6-fold higher than that in the Ti group. The BMP2 and VEGF concentrations in culture supernatants exhibited the following trends: Ti-Ca10Sr10, Ti-Ca10Sr5, and Ti-Ca10Sr2.5 > Ti-Sr10 and Ti-Ca10 > Ti; and Ti-Ca10Sr10 and Ti-Ca10Sr5 > Ti-Ca10Sr2.5 > Ti-Sr10 > Ti-Ca10 > Ti, respectively.

The expression levels of CCR7 (M1 marker), CD206 (M2 marker), BMP2, and VEGF in RAW264.7 cells were determined by RT-PCR (Figure 4B). CCR7 expression showed the following trend: Ti > Ti-Ca10 > Ti-Sr10 > Ti-Ca10Sr2.5 > Ti-Ca10Sr10 and Ti-Ca10Sr5. By contrast, CD206 and VEGF expression levels were 2.8- and 2.6-fold higher, respectively, in the Ti-Ca10Sr10 and Ti-Ca10Sr5 groups than in the Ti group. BMP2 expression exhibited the following trend: Ti-Ca10Sr5 > Ti-Ca10Sr10 > Ti-Ca10Sr2.5 > Ti-Sr10 > Ti-Ca10 > Ti.

**Figure 5:**
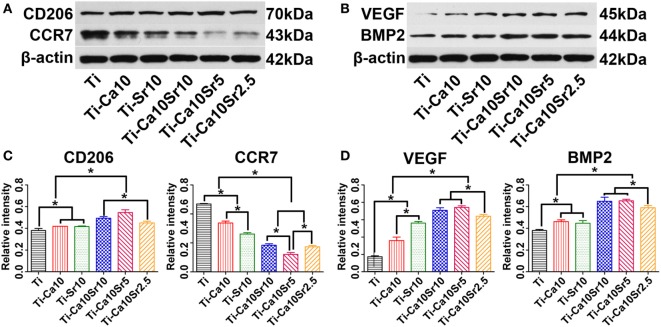
Western blotting analyses of proteins from RAW264.7 cells at day 4 of culture on different samples. **(A,B)** Western blotting images of proteins cluster of differentiation 206 (CD206), C-C chemokine receptor type 7 (CCR7), vascular endothelial growth factor (VEGF), and bone morphogenetic protein 2 (BMP2), respectively. **(C,D)** The corresponding gray values of the four proteins. *p < 0.05.

Cluster of differentiation 206 (M2 marker), CCR7 (M1 marker), VEGF, and BMP2 protein levels in RAW264.7 cells were determined by western blotting (Figures 5A,B), and quantified by image analysis (Figures 5C,D); the results were consistent with the RT-PCR analysis. CD206, VEGF, and BMP2 protein levels were 1.4-, 4.1-, and 1.7-fold higher in the Ti-Ca10Sr5 group than in the Ti group. By contrast, the CCR7 protein level was downregulated and exhibited the following trend: Ti > Ti-Ca10 > Ti-Sr10 > Ti-Ca10Sr2.5 and Ti-Ca10Sr10 > Ti-Ca10Sr5.

### *In Vitro* Responses of BMSCs in Co-Culture

**Figure 6:**
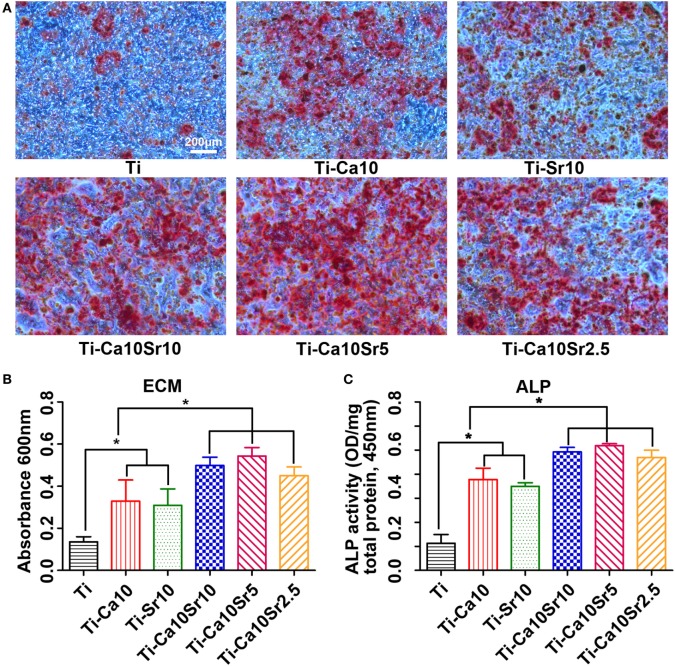
Responses of mouse BMSCs by co-culture with RAW264.7 cells which were pre-cultured for 4 days on different samples. **(A)** Extracellular matrix (ECM) areas of BMSCs determined using Alizarin red staining at day 21 of co-culture. Scale bar, 200 µm. **(B)** Optical density (OD) values of ECM eluted from BMSCs. **(C)** OD values of alkaline phosphatase activity (ALP) from BMSCs at day 14 of co-culture. *p < 0.05.

Extracellular matrix mineralization of co-cultured BMSCs was assessed by Alizarin red staining. The areas of ECM mineralization in the Ti-Ca10Sr10, Ti-Ca10Sr5, and Ti-Ca10Sr2.5 groups were larger than those in the Ti-Sr10, Ti-Ca10, and Ti groups (Figure 6A). The trend was as follows: Ti-Ca10Sr10, Ti-Ca10Sr5, and Ti-Ca10Sr2.5 > Ti-Sr10 and Ti-Ca10 > Ti (Figure 6B). The ALP activity of co-cultured BMSCs exhibited a similar trend (Figure 6C). The Ti-Ca10Sr5 group showed the highest ALP activity (2.59 ± 0.04 OD/mg total protein), compared to 2.46 ± 0.09, 2.35 ± 0.15, 1.75 ± 0.07, 1.71 ± 0.08, and 0.56 ± 0.18 for the Ti-Ca10Sr10, Ti-Ca10Sr2.5, Ti-Sr10, Ti-Ca10, and Ti groups, respectively.

**Figure 7:**
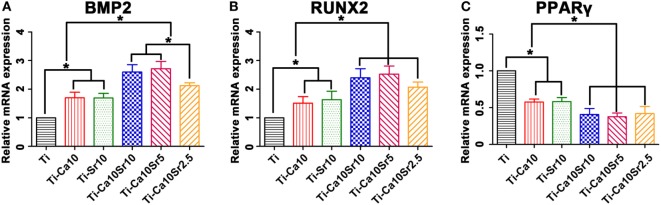
Osteogenic and adipogenic gene expression [bone morphogenetic protein 2 (BMP2), runt-related transcription factor 2 (RUNX2), and peroxisome proliferator-activated receptor γ (PPARγ)] of BMSCs at day 14 of co-culture with RAW264.7 cells which were pre-cultured for 4 days on different samples. **(A)** Expression of osteogenic gene BMP2. **(B)** Expression of osteogenic gene RUNX2. **(C)** Expression of adipogenic gene PPARγ. *p < 0.05.

The expression levels of the osteogenic genes BMP2 and RUNX2 and the adipogenic gene PPARγ in co-cultured BMSCs were determined by RT-PCR. BMP2 and RUNX2 expression was upregulated by the presence of Ca and Sr (Figure 7A). BMP2 expression levels were 2.12 ± 0.10, 2.72 ± 0.26, 2.60 ± 0.26, 1.69 ± 0.16, 1.70 ± 0.19, and 1.00 in the Ti-Ca10Sr10, Ti-Ca10Sr5, Ti-Ca10Sr2.5, Ti-Sr10, Ti-Ca10, and Ti groups, respectively. Similarly, RUNX2 expression in the Ti-Ca10Sr5 group was 2.5-fold higher than the Ti group (Figure 7B). Conversely, PPARγ expression was significantly downregulated in the Ti-Ca10Sr5 group and upregulated in the Ti group, and exhibited the following trend: Ti > Ti-Ca10 and Ti-Sr10 > Ti-Ca10Sr10, Ti-Ca10Sr5, and Ti-Ca10Sr2.5 (Figure 7C).

### *In Vivo* Responses of Macrophages

**Figure 8:**
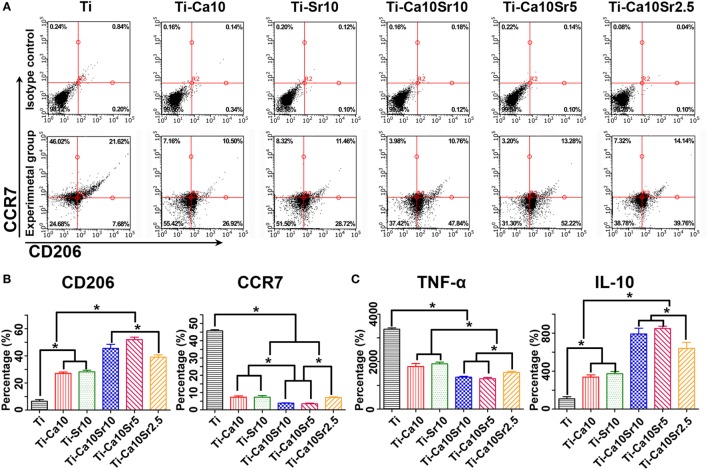
Flow cytometry analyses of cell-surface makers on macrophage and enzyme-linked immunosorbent assay determination of cytokines from the mouse air-pouch exudates. **(A)** Representative dot plots for expression of F4/80, C-C chemokine receptor type 7 (CCR7) and cluster of differentiation 206 (CD206) of macrophage surface makers. **(B)** Percentage of F4/80 positive cells expressing CD206 (M2 marker) and CCR7 (M1 maker), respectively. **(C)** The production of cytokines TNF-α and IL-10, respectively. *p < 0.05.

Air-pouch exudates were subjected to flow cytometry for F4/80 CCR7 and CD206 as markers of mouse macrophages, M1 macrophages, and M2 macrophages, respectively (Figures 8A,B). Compared with the Ti group, the Ti-Ca10Sr10 and Ti-Ca10Sr5 groups showed lower proportions of M1 macrophages, which exhibited the following trend: Ti > Ti-Ca10Sr2.5, Ti-Sr10, and Ti-Ca10 > Ti-Ca10Sr10 and Ti-Ca10Sr5. By contrast, the Ti-Ca10Sr5 group had the highest proportion of M2 macrophages, which showed the following trend: Ti-Ca10Sr5 > Ti-Ca10Sr10 > Ti-Ca10Sr2.5 > Ti-Sr10 and Ti-Ca10 > Ti.

Tumor necrosis factor-α and IL-10 concentrations in the air-pouch exudates were determined by ELISA (Figure 8C). The Ti-Ca10Sr5 group had a lower TNF-α concentration and a higher IL-10 concentration than the other five groups; this is in agreement with the *in vitro* ELISA results. The TNF-α and IL-10 concentrations showed the following trends: Ti > Ti-Sr10 and Ti-Ca10 > Ti-Ca10Sr2.5 > Ti-Ca10Sr10 and Ti-Ca10Sr5; and Ti-Ca10Sr10 and Ti-Ca10Sr5T > Ti-Ca10Sr2.5 > Ti-Sr10 and Ti-Ca10 > Ti, respectively.

**Figure 9:**
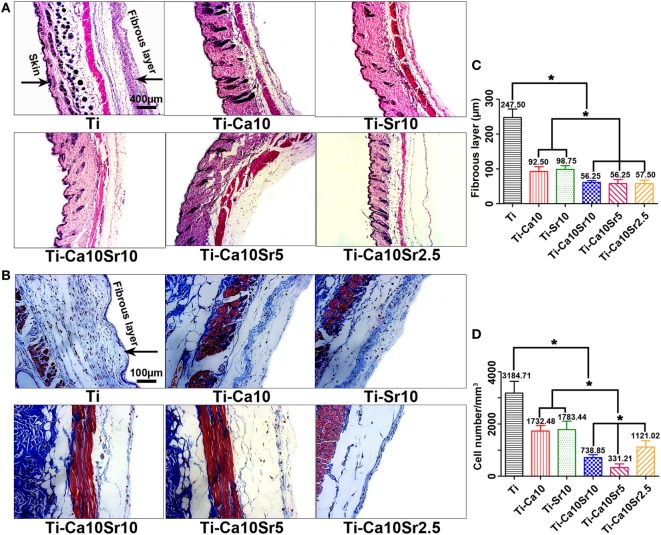
Images of hematoxylin eosin (HE) staining and Masson’s trichrome staining on the air-pouch tissues adjacent to different samples at day 7 of implantation. **(A)** HE stained images showing a whole structure and layers of the air-pouch tissues. Scale bar, 400 µm. **(B)** Masson’s trichrome stained images obviously displaying the fibrous tissues and the infiltration cells. Scale bar, 100 µm. **(C)** Thickness of fibrous layer. **(D)** Numbers of infiltration cells. *p < 0.05.

Air-pouch tissue sections were stained with HE and Masson’s trichrome (Figures 9A,B). The fibrous capsule around the air-pouch tissue was thinner in the presence of both Ca and Sr (Figure 9C). The thickness of the fibrous layer in the Ti-Ca10Sr5, Ti-Ca10Sr10, and Ti-Ca10Sr2.5 groups was 56.25 ± 7.66, 56.25 ± 9.88, and 57.50 ± 10.27 µm, respectively. This was in agreement with the trend in the number of infiltrating inflammatory cells: Ti > Ti-Ca10 and Ti-Sr10 > Ti-Ca10Sr2.5 > Ti-Ca10Sr10 > Ti-Ca10Sr5 (Figure 9D).

**Figure 10:**
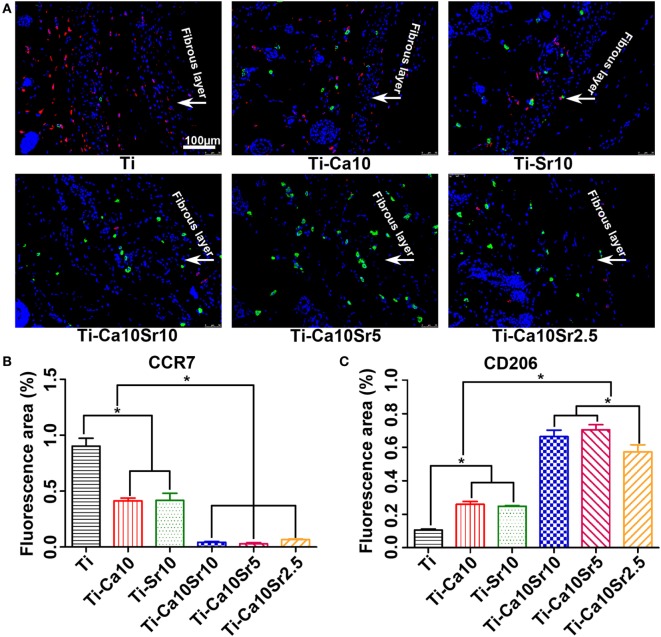
Images of immunofluorescence staining on air-pouch tissues adjacent to different samples at day 7 of implantation. **(A)** Immunofluorescence stained images showing two phenotypes of macrophage polarization. C-C chemokine receptor type 7 (CCR7) (M1 marker) positive cells were stained in red and cluster of differentiation 206 (CD206) (M2 marker) positive cells in green. Scale bar, 100 µm. **(B,C)** Percentage of corresponding fluorescence area representing CCR7- and CD206-positive regions, respectively. *p < 0.05.

Air-pouch tissue sections were also subjected to immunofluorescence staining for surface markers of M1 (red; CCR7) and M2 macrophages (green; CD206) (Figure 10A). The CCR7- and CD206-positive areas are shown in Figures 10B,C, respectively. The CCR7-positive area was largest in the Ti group (0.90 ± 0.069) and smallest in the Ti-Ca10Sr5 group (0.03 ± 0.009). By contrast, the CD206-positive area was 0.71 ± 0.036, 0.66 ± 0.037, 0.57 ± 0.037, 0.26 ± 0.015, 0.25 ± 0.003, and 0.11 ± 0.006 in the Ti-Ca10Sr5, Ti-Ca10Sr10, Ti-Ca10Sr2.5, Ti-Ca10, Ti-Sr10, and Ti groups, respectively.

## Discussion

Calcium and Sr can induce osteogenesis and suppress inflammation (34), but the role of the Ca:Sr ratio in osteoimmunomodulation is unclear. Indeed, different Ca:Sr ratios could exert synergistic and/or competitive effects. MAO is commonly used to modify implant surfaces with the aim of inducing suitable biological responses, such as improved osseointegration (35–38). In this work, to increase osteoinductive activity, Ca and/or Sr-doped Ti-oxide coatings were fabricated on a Ti substrate by MAO and ECT. The results suggest that the Ca:Sr concentration ratio influences macrophage polarization, and coating Ti with Ca and Sr at a 2:1 ratio enhanced osteoimmunomodulation.

Macrophages play an important role in the immune response to implanted biomaterials. In response to environmental signals, macrophages differentiate into either the M1 or M2 phenotype. M1 macrophages produce pro-inflammatory cytokines (e.g., TNF-α, IL-6), and M2 macrophages secrete anti-inflammatory cytokines (e.g., IL-10, IL-1ra), which enhance angiogenesis and tissue repair (39–42). The *in vitro* and *in vivo* results showed that Ca and/or Sr significantly induced M2 macrophage polarization, which resulted in increased production of IL-10. In addition, Ti coated with both Ca and Sr, particularly at a 2:1 ratio, induced macrophage polarization toward the M2 phenotype. This resulted in increased BMP2, VEGF, and IL-10 production; these factors contribute to bone formation, angiogenesis, and tissue repair.

Brown et al. suggested that major failures of medical implants, such as loosening and erosion, are attributable to an inflammatory reaction, which results in inflammatory cell infiltration and formation of a thick fibrous capsule around the biomaterial (43). This capsule provides a niche within which pathogens are concealed from the immune response (44). In this study, coating of Ti with Ca and Sr at a 2:1 ratio significantly promoted M2 macrophage polarization, reduced inflammatory cell infiltration, inhibited fibrous capsule formation, and increased production of BMP2 and VEGF. Therefore, Ca- and Sr-coated Ti shows promise for use in implanted biomaterials. Moreover, M2 macrophages reportedly inhibit the inflammatory response to biomaterials and enhance tissue regeneration and binding of the biomaterial implant to host tissue (45, 46).

Strontium and Ca have similar chemical and biological properties, and both are indispensable for humans (34). These two elements exert synergistic effects in certain biological processes. For example, Ca and Sr are regulators and agonists of Ca-sensing receptors (47). Bone cells express receptors to promote the differentiation, proliferation, and mineralization of BMSCs (48, 49). However, Ca acts as a competitive inhibitor of Sr influx, and Sr inhibits uptake of Ca by suppressing the mitochondrion membrane potential (ΔΨ)-modulated efflux pathway (23, 50–52). In this study, the Ca and Sr concentration ratios in the Ti-Ca10Sr10, Ti-Ca10Sr5, and Ti-Ca10Sr2.5 groups were gradually and approximately kept at 1:1, 2:1, and 4:1, respectively, with increasing duration of immersion. Furthermore, coating of the Ti surface with 10% Ca and 5% Sr enhanced the synergistic effect and weakened the competitive effect on macrophage polarization toward the M2 phenotype, and enhanced the osteoimmunomodulatory activity. This effect may also be due to the higher surface wettability of the 2:1 Ca:Sr coating, which enhances protein adsorption and cell adhesion (53, 54). Therefore, a Ca:Sr ratio of 2:1 is optimum in terms of enhancing macrophage polarization toward the M2 phenotype. However, the underlying mechanism is unclear and so further research is warranted.

The results support our hypothesis that a certain Ca:Sr ratio would be optimum in terms of macrophage polarization toward the M2 phenotype. M2 phenotype polarization was greatest in the Ti-Ca10Sr5 group, which resulted in increased production of osteogenic growth factors, such as BMP2 and VEGF. These growth factors play an important role in osteogenic differentiation of BMSCs. For example, VEGF induces angiogenesis, which facilitates bone regeneration by enhancing transport of nutrients and oxygen (55). BMP2 facilitates new bone formation by promoting the osteogenic differentiation of BMSCs (56, 57). The osteogenic effects of M2 macrophages were demonstrated by the BMP2, RUNX2, and PPARγ expression levels of BMSCs co-cultured with RAW264.7 cells pre-cultured on Ca/chromium-coated Ti substrates. As an adipogenic gene, PPARγ suppresses osteogenic differentiation of BMSCs and acts as an antagonist of BMP2 and RUNX2 (58, 59). This is consistent with previous reports that bone healing, which is regulated by BMSCs, is influenced by macrophage phenotype (43, 55).

Co-culture techniques are used to mimic *in vivo* conditions (60). In this work, after 3 days of culture on the sample surfaces, RAW264.7 cells were co-cultured with BMSCs. This prevents any effect of Ca and/or Sr on BMSC differentiation, and allows free exchange of soluble factors (61). Therefore, biomaterial-induced macrophage polarization and the resulting osteogenic effects can be mimicked *in vitro* by co-culture. Ti coated with Ca and Sr at a 2:1 ratio was optimal in terms of inducing macrophage polarization toward the M2 phenotype. Furthermore, after pre-culture on the Ti surface doped with a Ca/Sr ratio of 2:1, RAW264.7 cells enhanced the osteogenic differentiation of BMSCs. This is supported by the results of the ECM mineralization and ALP assays.

However, this work is limited by insertion of the material samples into mouse bone-marrow cavities to evaluate osseointegration between the implant and the host bone. The club-shaped materials subjected to MAO and ECT could not be implanted because the bone-marrow cavities are smaller than the diameter of the materials. In addition, whether osseointegration between the material and the host bone was due to macrophage polarization or a direct effect of Ca and/or Sr could not be determined because Ca and/or Sr themselves induce osteogenic differentiation of BMSCs (62, 63). The *in vivo* air-pouch model and *in vitro* co-culture showed that the coated Ti materials induced macrophage polarization toward the M2 phenotype, increased production of BMP2 and VEGF, and enhanced BMSC osteogenic differentiation.

In this work, Ca and/or Sr-doped Ti-oxide coatings were fabricated on a Ti substrate. The results showed that the Ca:Sr concentration ratio influences macrophage polarization. Ti coated with Ca and Sr at a 2:1 ratio resulted in the greatest M2 polarization *in vitro* and *in vivo* and enhanced the osteogenic differentiation of BMSCs. Our findings will facilitate the design of immunomodulatory coatings that enhance osseointegration of orthopedic implants.

## Ethics Statement

Pathogen-free C57BL/6 mice (male, aged 6 weeks) used for *in vitro* and *in vivo* experiments were kept under specific pathogen-free conditions at our animal care facility. Animal maintenance and experiments were conducted according to the policy of the Institutional Animal Care and Use Committee of Shanghai Jiao Tong University, the regulations for the Administration of Affairs Concerning Experimental Animals (China, 2014), and the National Institutes of Health Guide for the Care and Use of Laboratory Animals (GB14925-2010). All animal experiments in this work were approved by the Animal Care and Experiment Committee of Shanghai Sixth People’s Hospital affiliated with Shanghai Jiao Tong University.

## Author Contributions

XY, HC, XZ, and XL designed the study. XY, KT, and HC performed the study. XY performed statistical analysis and drafted the manuscript with HC, BL, JW, YZ, MC, and HQ helped revise the manuscript. All authors read and approved the final manuscript.

## Conflict of Interest Statement

All authors declared that the research was conducted in the absence of any commercial or financial relationships that could be construed as a potential conflicted of interest.

## Supplementary Material

Supplementary material related to this article can be found. Schematic illustration about this work is showed in *Graphic for Abstract*. As showed in Figure S2 in Supplementary Material, there is no significant difference in body temperature from the six experimental groups at day 0, 1, 4, and 7 after surgery. Figures S3–S6 in Supplementary Material, respectively, make clear the histomorphology of mouse heart, liver, spleen, and kidney for the six experimental groups. No abnormal tissues and obvious infiltration of inflammatory cells were evidenced. These results demonstrated that the material concerned in this work probably stimulated little side effects to mice used in our experiments.
