# Supplementary material for: Immunomodulatory Effects of Calcium and Strontium Co-Doped Titanium Oxides on Osteogenesis
Source: Front Immunol. 2017 Sep 29;8:1196. doi: 10.3389/fimmu.2017.01196 (PMC5626827; doi:10.3389/fimmu.2017.01196)
Supplement: Supplementary file 1 — Supplementary material related to this article can be found. Schematic illustration about this work is showed in Graphic for Abstract. As showed in Figure S2 in Supplementary Material, there is no significant difference in body temperature from the six experimental groups at day 0, 1, 4, and 7 after surgery. Figures S3–S6 in Supplementary Material, respectively, make clear the histomorphology of mouse heart, liver, spleen, and kidney for the six experimental groups. No abnormal tissues and obvious infiltration of inflammatory cells were evidenced. These results demonstrated that the material concerned in this work probably stimulated little side effects to mice used in our experiments. [file Data_Sheet_1.DOCX]

**Supplementary Material**

**Immunomodulatory Effects of Calcium and Strontium Co-Doped Titanium Oxides on Osteogenesis**

Xiangwei Yuan^1^, Huiliang Cao^2,*^, Jiaxing Wang^1^, Kaiwei Tang^2^, Bin Li^1^, Yaochao Zhao^1^, Mengqi Cheng^1^, Hui Qin^1^, Xuanyong Liu^2,*^, Xianlong Zhang^1,*^

^1^Department of Orthopedics, Shanghai Sixth People’s Hospital, Shanghai Jiao Tong University, Shanghai 200233, China

^2^State Key Laboratory of High Performance Ceramics and Superfine Microstructure, Shanghai Institute of Ceramics, Chinese Academy of Sciences, Shanghai 200050, China

*Corresponding authors: Xianlong Zhang (dr_zhangxianlong@sina.com), Xuanyong Liu (xyliu@mail.sic.ac.cn), Huiliang Cao ([hlc@mail.sic.ac.cn)](mailto:hlc@mail.sic.ac.cn))


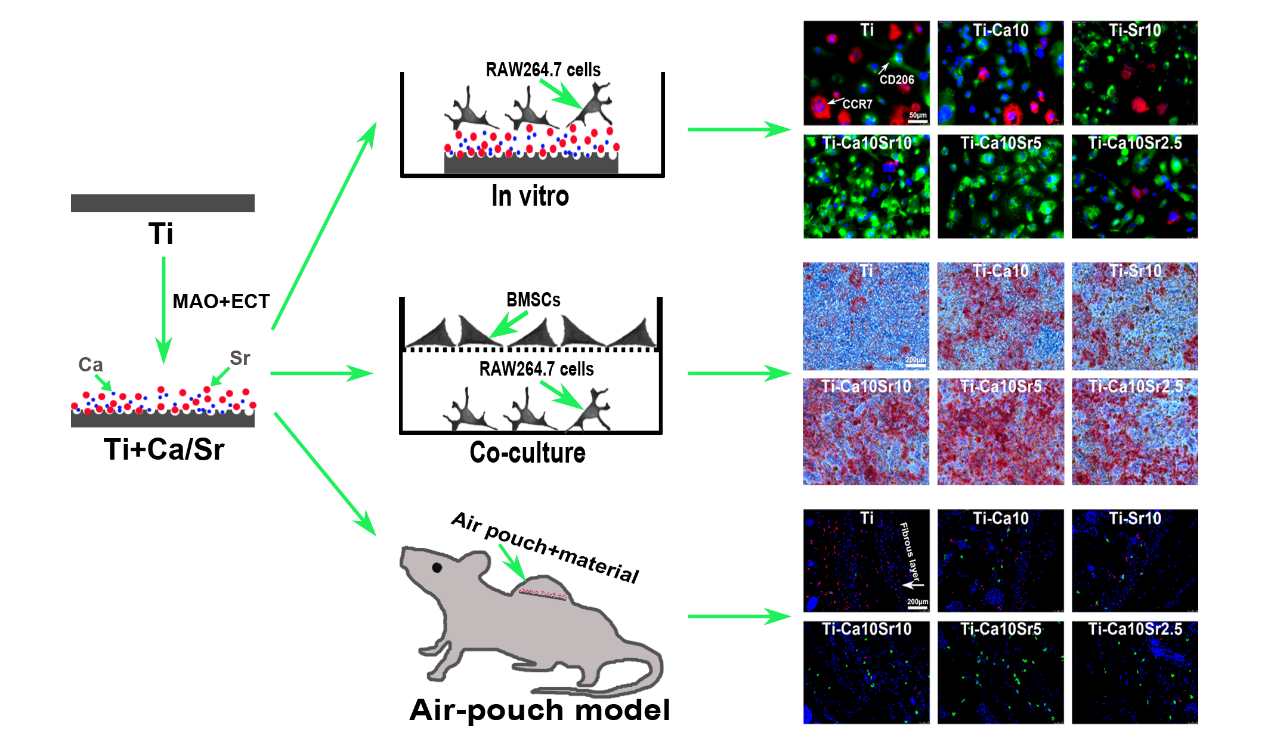


**Graphic for Abstract**. Schematic illustration about effects of Ca/Sr ratio on osteoimmunomodulation of biomaterials. In vitro and in vivo experiments proved the concentration ratio of Ca and Sr is a key factor affecting the polarization of macrophage, and the coating with a Ca/Sr ratio of 2:1 was better than others with various Ca or/and Sr concentrations in modulating M2 polarization, which contributes to the osteogenic differentiation of mouse bone marrow stromal cells (BMSCs) by co-culture with macrophages.


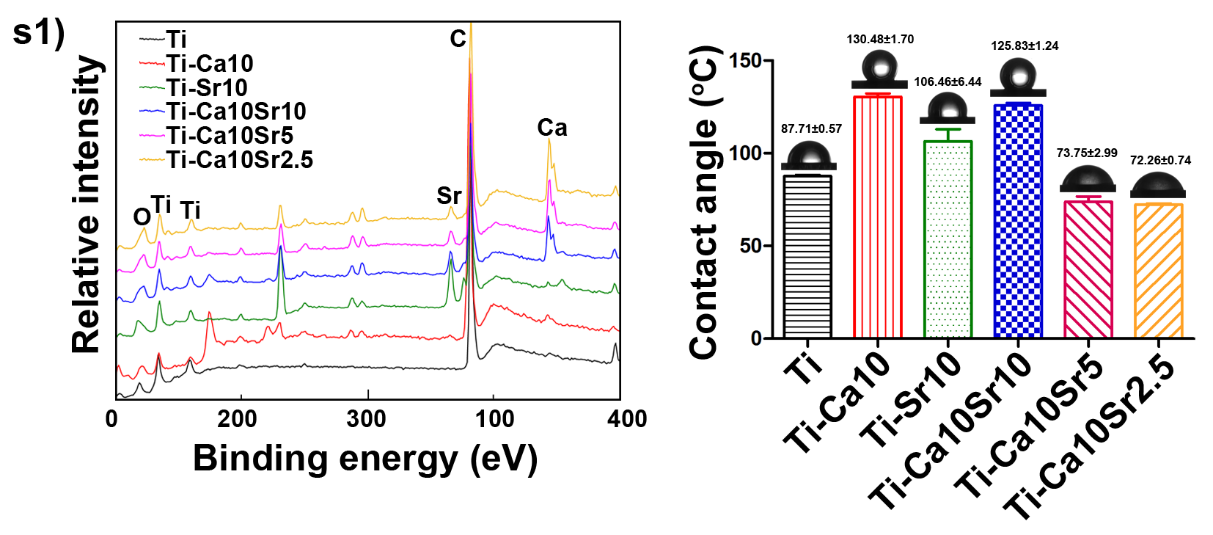


**Figure S1.** X-ray photoelectron spectroscopy (XPS) spectra of different samples (**the left figure**). Based on the peaks of Ca and Sr, the surface chemistry of samples was changed in the six groups which provided an additional evidence for the coatings with different Ca/Sr concentration ratios. Water contact angles on different sample surfaces (**the right figure**).  The Ti-Ca10 group, the most hydrophobic among the six groups, has water contact angles of 130.48±1.70, which was apparently reduced as the ratio of Ca/Sr was increased (the contact angle for the Ti-Ca10Sr10, Ti-Ca10Sr5, and Ti-Ca10Sr2.5 group is 125.83±1.24, 73.75±2.99, 72.26±0.74, respectively).


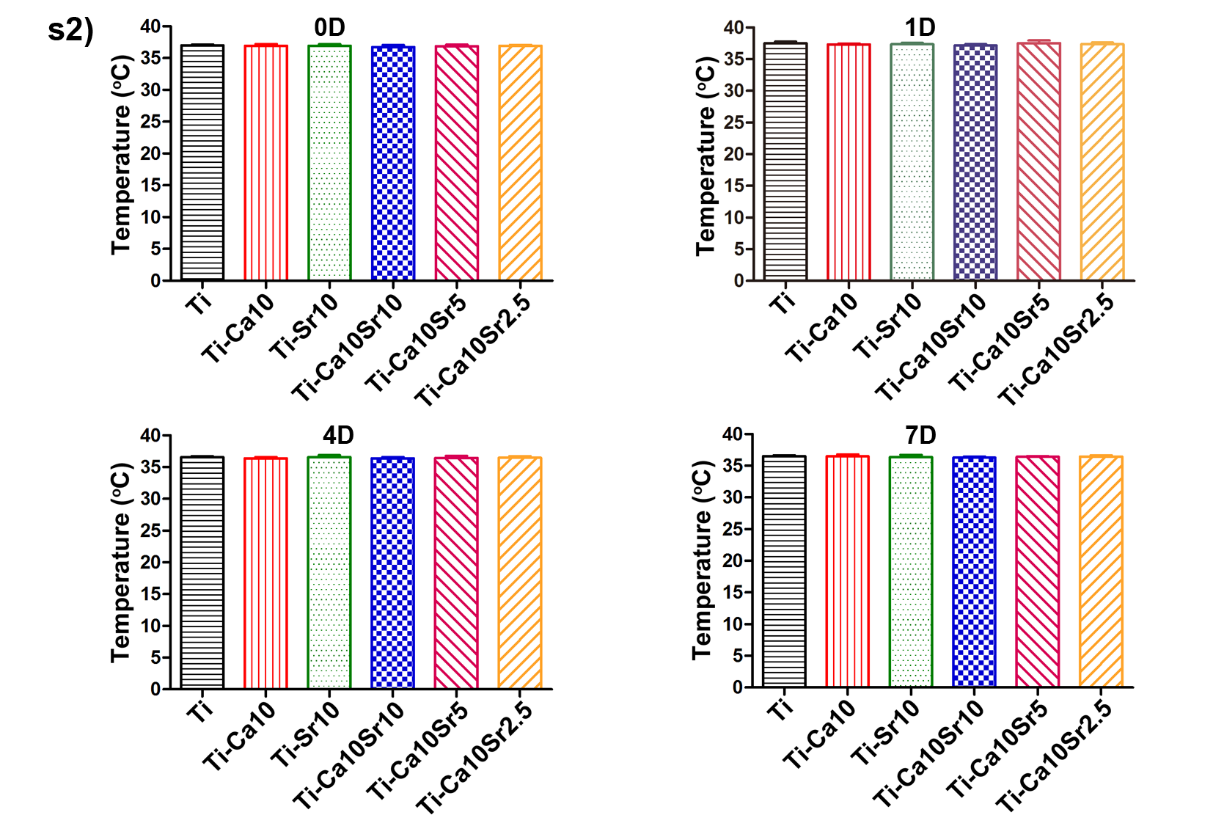


**Figure S2.** At day 0, 1, 4, and 7 after surgery, the rectal body temperature of mice was measured using a digital thermometer. No significant difference in temperature was evidenced among the six groups.


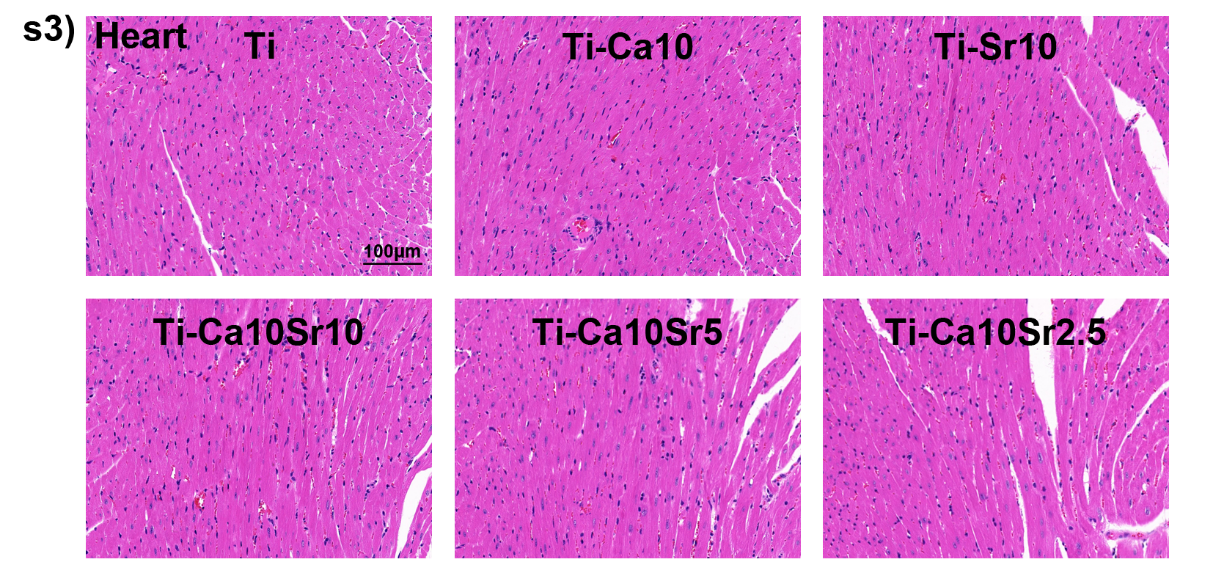


**Figure S3.** Hematoxylin eosin (HE) stained mouse myocardia at day 7 after surgery. The myocardial structure is clear in the six groups. Cardiac muscles appear longitudinal and horizontal among which blood vessels distribute. No infiltration of inflammatory cells was evidenced.


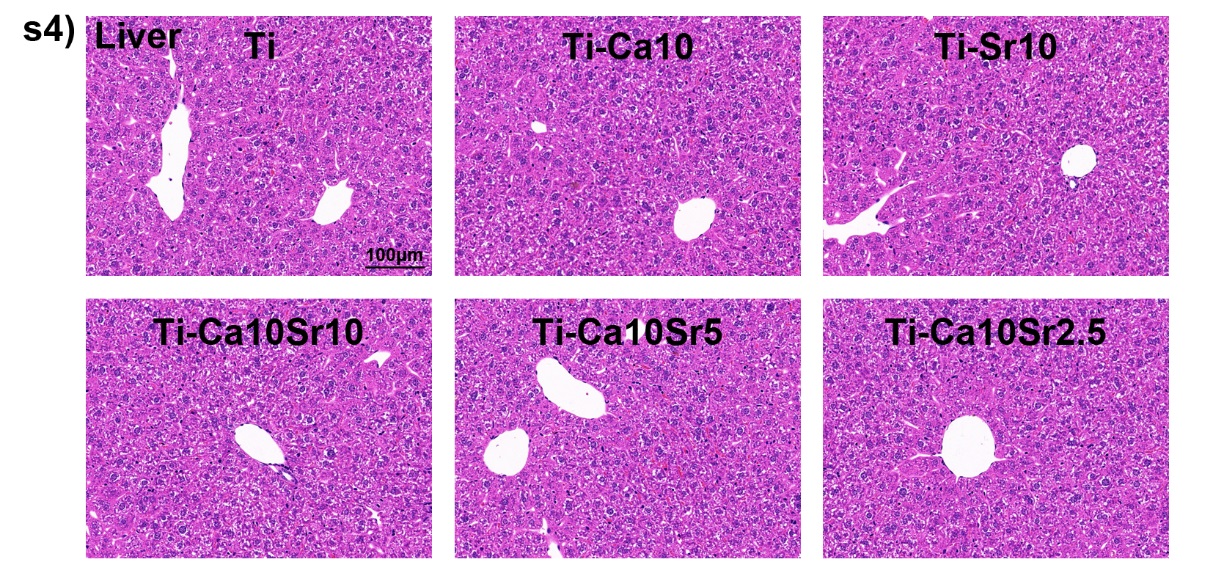


**Figure S4.** Hematoxylin eosin (HE) stained mouse livers at day 7 after surgery. As shown in the images, the central veins are clear in all the six groups, and the hepatocytes are arranged evenly accompanied by some Kupffer cells. In addition, the hepatocytes are uniform in size without degeneration, necrosis, and small number of inflammatory cells was evidence in all the six groups.


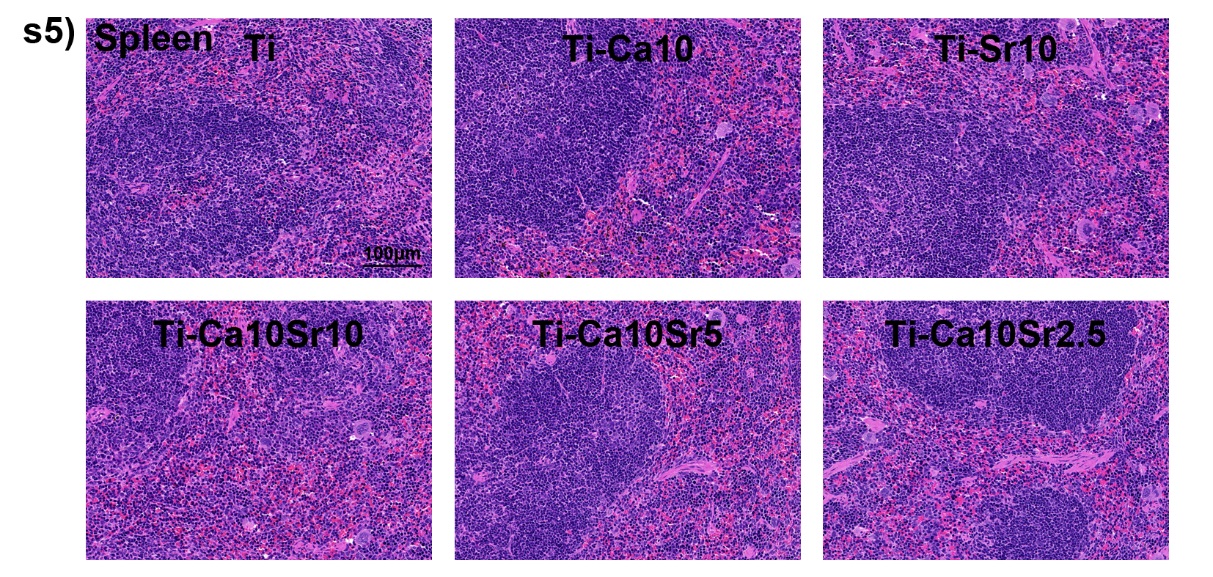


**Figure S5.** Hematoxylin eosin (HE) stained mouse spleens at day 7 after surgery. The spleen tissues are normal, consisting of splenic nodules and red pulps in all the six groups. And there are a few macrophages dispersing in splenic nodules of each group.


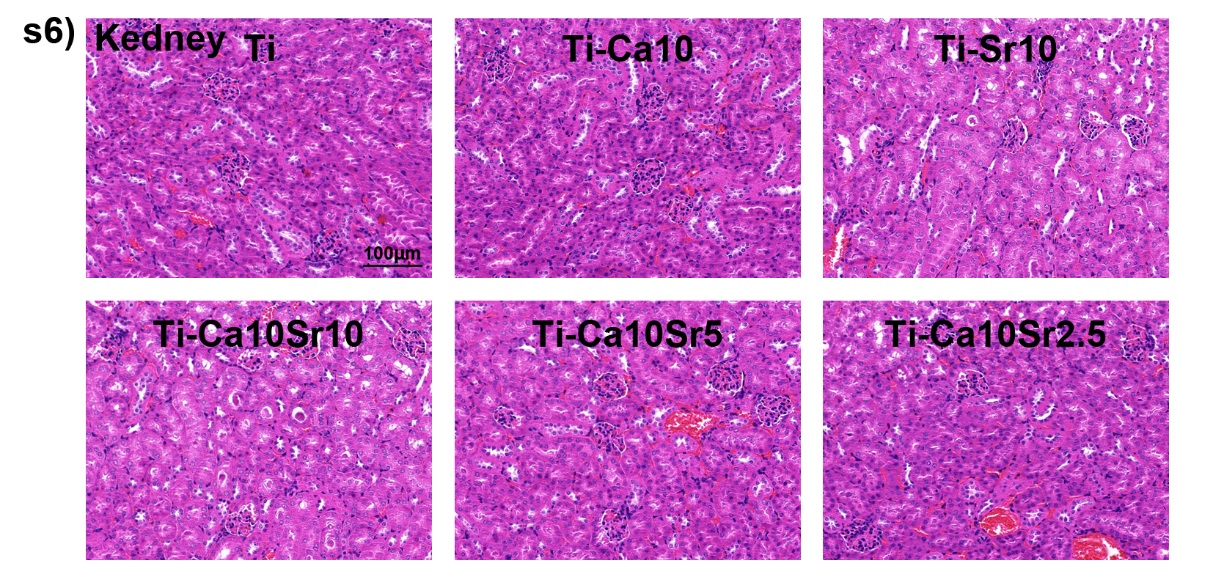


**Figure S6.** Hematoxylin eosin (HE) stained mouse kidneys at day 7 after surgery. The nephridial tissues are normal in all the six groups. There are glomeruli and renal tubules accompanied with renal arterioles. No obvious infiltration of inflammatory cells was evidenced.
